# Carbon dioxide inhibits COVID-19-type proinflammatory responses through extracellular signal-regulated kinases 1 and 2, novel carbon dioxide sensors

**DOI:** 10.1007/s00018-021-04005-3

**Published:** 2021-11-06

**Authors:** Hanna Galganska, Wieslawa Jarmuszkiewicz, Lukasz Galganski

**Affiliations:** 1 Molecular Biology Techniques Laboratory, Faculty of Biology, Adam Mickiewicz University, Uniwersytetu Poznanskiego 6, 61-614 Poznan, Poland; 2 Laboratory of Mitochondrial Biochemistry, Department of Bioenergetics, Faculty of Biology, Adam Mickiewicz University, Uniwersytetu Poznanskiego 6, 61-614 Poznan, Poland

**Keywords:** ERK1, ERK2, Interleukin-6, Hydrogen peroxide, Bicarbonate, S protein

## Abstract

Mitogen-activated protein kinase (MAPK) signalling pathways are crucial for developmental processes, oncogenesis, and inflammation, including the production of proinflammatory cytokines caused by reactive oxygen species and upon severe acute respiratory syndrome coronavirus 2 (SARS-CoV-2) infection. There are no drugs that can effectively prevent excessive inflammatory responses in endothelial cells in the lungs, heart, brain, and kidneys, which are considered the main causes of severe coronavirus disease 2019 (COVID-19). In this work, we demonstrate that human MAPKs, i.e. extracellular signal-regulated kinases 1 and 2 (ERK1/2), are CO_2_ sensors and CO_2_ is an efficient anti-inflammatory compound that exerts its effects through inactivating ERK1/2 in cultured endothelial cells when the CO_2_ concentration is elevated. CO_2_ is a potent inhibitor of cellular proinflammatory responses caused by H_2_O_2_ or the receptor-binding domain (RBD) of the spike protein of SARS-CoV-2. ERK1/2 activated by the combined action of RBD and cytokines crucial for the development of severe COVID-19, i.e. interferon-gamma (IFNγ) and tumour necrosis factor-α (TNFα), are more effectively inactivated by CO_2_ than by dexamethasone or acetylsalicylic acid in human bronchial epithelial cells. Previously, many preclinical and clinical studies showed that the transient application of 5–8% CO_2_ is safe and effective in the treatment of many diseases. Therefore, our research indicates that CO_2_ may be used for the treatment of COVID-19 as well as the modification of hundreds of cellular pathways.

**Supplementary Information:**

The online version contains supplementary material available at 10.1007/s00018-021-04005-3.

## Introduction

The physiological importance of carbon dioxide (CO_2_) in tissues, organs and the body is well understood. However, the influence of this common gas on the functioning and components of cells is poorly understood, even though all cells in which organic substances are oxidised in the processes of oxidative cellular respiration produce CO_2_. While CO_2_, as a small molecule, can be efficiently transported through membranes by diffusion, CO_2_ is spontaneously hydrated to HCO_3_^−^ at the pH of the cytoplasm. Carbonic anhydrases can convert HCO_3_^−^ to CO_2_ and vice versa. The widespread presence of carbonic anhydrases in bacteria, archaea and eukaryotes and the existence of additional CO_2_ and HCO_3_^−^ transporters indicate the benefits of rapid regulation of CO_2_ levels in cells, further suggesting the importance of CO_2_ for cell functions. It is, therefore, expected that there are mechanisms of CO_2_ sensing that are common to different groups of organisms. However, conserved CO_2_ receptors have not yet been found, and the currently identified eukaryotic CO_2_ sensors are specific to only particular groups of organisms, e.g. insect chemoreceptors. In mammals, several families of proteins are considered HCO_3_^−^ receptors, including protein tyrosine phosphatases, which are inactivated by HCO_3_^−^-assisted oxidation using H_2_O_2_ [1], adenylyl cyclases (ACs) [2] and guanylyl cyclases (GCs) [3].

Mitogen-activated protein kinases (MAPKs) have been found in all eukaryotes. They regulate many aspects of cell survival, proliferation, differentiation, migration, apoptosis, neurodegeneration and oncogenesis. Typically, MAPKs transduce signals from receptors to a wide variety of effector proteins, including transcription factors. Several human MAPKs, including c-Jun N-terminal kinase (JNK), extracellular signal-regulated kinases 1 and 2 (ERK1/2) and p38, are involved in hundreds of documented signalling pathways in all organs, tissues, and cell types. JNK, ERK1/2 and p38 are activated through phosphorylation at regulatory tyrosine and threonine residues in the activation loop by upstream MAPK kinases (MEKs) and dephosphorylated and inactivated by MAPK phosphatases (MKPs). MAPKs are key players in the regulation of inflammation. In response to a wide variety of chemical and biological agents, such as reactive oxygen species (ROS), they regulate the production of ROS and proinflammatory cytokines, including interferon-gamma (IFNγ), interleukin-1β, interleukin-6 (IL-6) and tumour necrosis factor-α (TNFα) [4, 5].

Angiotensin-converting enzyme 2 (ACE2) is a potent inhibitor of MAPK signalling and thus efficiently prevents both activation of MAPKs and pneumonia caused by exposure to cigarette smoke, particulate matter 2.5 (PM2.5), lipopolysaccharides (LPS) and bleomycin [6, 7]. However, disruption of ACE2 function occurs during severe acute respiratory syndrome coronaviruses 1 and 2 (SARS-CoV and SARS-CoV-2, respectively) infection, when the ACE2 receptor is bound by viral spike protein leading to MAPK activation and the production of proinflammatory cytokines and causing pneumonia or even acute respiratory distress syndrome (ARDS) [8]*.* Therefore, the inhibition of active MAPKs could be a strategy to prevent acute severe coronavirus disease 2019 (COVID-19).

Since inactive plant MAPKs are activated upon CO_2_ binding, and active MAPKs are inactivated by CO_2_ [9], we investigated MAPK susceptibility to CO_2_ in vitro and in human cells.

## Materials and methods

### Cell culture

The stable human umbilical vein endothelial cell (EC) line EA.hy926 (ATCC CRL-2922, RRID:CVCL_3901, ATCC, Manassas, Virginia, US) and normal human bronchial epithelial cell line BEAS-2B (ATCC CRL-9609, RRID:CVCL_UR57) were cultured in Dulbecco’s modified Eagle’s medium (DMEM; with 5.55 or 25 mM glucose) or DMEM/nutrient mixture F-12 (DMEM/F12; 17.5 mM glucose), respectively, supplemented with heat-inactivated 10% foetal bovine serum (FBS), 1% l-glutamine and 1% penicillin/streptomycin. The EA.hy926 cell culture medium was additionally supplemented with 2% hypoxanthine-aminopterin-thymidine (HAT). The cells were cultured in a humidified 5% CO_2_ atmosphere at 37 °C until they reached approximately 80% (BEAS-2B) or 95% (EA.hy926) confluence, and the medium was exchanged every 3 days. The cells were treated with the indicated concentrations of the receptor-binding domain (RBD) of SARS-CoV-2 spike protein (ABclonal, RP01278) and dexamethasone (dex) (Sigma-Aldrich, D1756), 1 mg/ml LPS (Cell Signaling Technology, #14011), 10 ng/ml TNFα (Cell Signaling Technology, #8902SC), 7.5 ng/ml IFNγ (Merck, IF002), 5 mM (E/Z)-BCI hydrochloride (BCI; Sigma-Aldrich, B4313), 2.5 µM U0126 (Adooq Bioscience, A10957), 25 µM PD98059 (Adooq Bioscience, A10705) and phosphate-buffered saline (PBS) and/or dimethyl sulfoxide (DMSO) as controls 2 days after the cells had reached confluence and 12 h (for treatments shorter than 1 h) or 2 h (for longer treatments) after medium exchange.

Primary cells, i.e. human umbilical vein endothelial cells (HUVECs, Lonza, C2519A) and human pulmonary microvascular endothelial cells (HPMECs, ScienCell, 3000), were cultured in endothelial cell growth medium-2 (EGM-2, Lonza, CC-3162) or microvascular endothelial cell growth medium-2 (EGM-2 MV, Lonza, CC-3202), respectively, according to the manufacturers’ protocols. The HPMECs were grown on vessels coated with 2 μg/cm^2^ bovine plasma fibronectin (ScienCell, 8248). All experiments were conducted at passages 2–3 using confluent HUVECs and HPMECs at 70–80% confluency after exchange of EGM-2 or EGM-2 MV with heparin- and hydrocortisone-free media. A number of administered spike-pseudotyped lentivirus particles corresponded to the doubled tissue culture infectious dose 50% (TCID_50_) in a standard assay (2 days at 37 °C with 5% CO_2_) on HEK293 cells expressing ACE2.

The following reagents were deposited by the Centers for Disease Control and Prevention and obtained through BEI Resources, NIAID, NIH: SARS-Related Coronavirus 2, Isolate USA-WA1/2020, heat inactivated (NR-52286) and SARS-Related Coronavirus 2, Isolate USA/CA_CDC_5574/2020, heat inactivated, NR-55245. The following reagents were obtained through BEI Resources, NIAID, NIH: SARS-Related Coronavirus 2, Wuhan-Hu-1 spike-pseudotyped lentivirus, Luc2/ZsGreen, NR-53818, SARS-Related Coronavirus 2, Wuhan-Hu-1 spike D614G-pseudotyped lentivirus, Luc2/ZsGreen, NR-53819, and Spike glycoprotein (stabilised) from SARS-Related Coronavirus 2, B.1.1.7 lineage with C-terminal histidine and Avi tags, recombinant from HEK293 cells, NR-55311.

### General considerations regarding CO_2_ treatment

The CO_2_ concentrations (6.5–10%) used for ERK1/2 regulation are in the physiological range based on the following assumptions. (i) In central venous blood, the normal partial pressure of CO_2_ (pCO_2_), 45 mm Hg [10], is equal to both 6 kPa and CO_2_ dissolved from an atmosphere containing 6% CO_2_. (ii) Typical therapeutic breathing of 5% CO_2_ (see **“**Discussion**”**) leads to an increase in the blood pCO_2_ by 10–13 mm Hg. After summing (58 mm Hg), the pCO_2_ corresponds to dissolved CO_2_ from air containing 8% CO_2_. (iii) The intracellular pCO_2_ is higher than that in blood. CO_2_/HCO_3_^−^ calculations were made using Aqion software (www.aqion.de).

### Immunoblotting

Immunoblotting was carried out as described previously [11]. The following antibodies were purchased from Abcam: anti-GAPDH (1:2500, 37 kDa, AB9485, RRID:AB_307275), anti-IL-6 (1:2000, 17 and 50 kDa, ab9324, RRID:AB_307175), and anti-ICAM-1 (1:4000, 90 kDa, AB53013, RRID:AB_870702). Anti-ACE2 (1:1000, 125 kDa, MA532307, RRID:AB_2809589) and anti-HIF-1α (1:1000, 130 kDa, PA5-85,494, RRID:AB_2792634) antibodies were obtained from Thermo Scientific. The following antibodies were purchased from Cell Signaling Technology: anti-ERK1/2 (1:1000, 42, 44 kDa, #4695, RRID:AB_390779), anti-phospho-ERK1/2 (Thr202/Tyr204), (1:1000, 42, 44 kDa, # 9101, RRID:AB_331646), anti-MSK1 (1:1000, 95 kDa, #3489, RRID:AB_2285349), anti-phospho-MSK1 (Thr581) (1:1000, 95 kDa, #9595, RRID:AB_2181783), anti-p38 (1:1000, 43 kDa, #9212, RRID:AB_330713), anti-phospho-p38 (Thr180/Tyr182) (1:1000, 43 kDa, #4511, RRID:AB_2139682), anti-phospho-JNK (Thr183/Tyr185) (1:1000, 46, 54 kDa, #4668, RRID:AB_823588), anti-JNK (1:1000, 46, 64 kDa, #9252, RRID:AB_2250373), anti-phospho-NFκB p65 (Ser536) (1:1000, 70 kDa, #3033, RRID:AB_331284), and anti-NFκB p65. (1:1000, 70 kDa, #6956, RRID:AB_10828935). The following antibody were obtained from ABclonal (Woburn, MA): anti-phospho-NFkB p65 (S276) (1:1000, 70 kDa, AP0123, RRID:AB_2771505). An anti-phospho-MBP antibody (1:200, 18 kDa, 13–104) was purchased from Merck.

### Immunoprecipitation

ECs were rinsed twice with ice-cold PBS, scraped off and centrifuged (700×*g*, 1 min, 4 °C). The pellet containing 2 × 10^8^ ECs was lysed (5 min on ice) in 2 ml of buffer containing 100 mM Tris–HCl pH 7.5, 600 mM NaCl, 75 mM NaF, 6 mM EDTA, 0.1% NP40, 4 mM DTT, 1 mM PMSF and protease (Roche) and phosphatase (Thermo Scientific) inhibitor cocktails. Then, 5 ml of ice-cold deionised water was added, and the lysates were centrifuged (10,000×*g*, 10 min, 4 °C). The supernatants were incubated for 1 h at 4 °C with 250 μl of Dynabeads^®^ Protein G (Life Technologies, Oslo, Norway) for preclearing. Then, the Dynabeads^®^ Protein G was replaced with 40 ml of anti-ERK1/2 antibody coupled with Dynabeads. The suspensions were incubated overnight at 4 °C with slow agitation and then washed six times with PBST and two times with deionised water.

### ERK1/2 dephosphorylation

Immunoprecipitated ERK1/2 were dephosphorylated at 37 °C for 1 h in a reaction mixture containing 10 mM Tris–HCl pH 8.0, 5 mM MgCl_2_, 100 mM KCl, 0.02% Triton X-100 and 600 U of alkaline phosphatase (FastAP, Thermo Scientific). After FastAP inactivation with 50 mM EDTA followed by six washes with PBST and two washes with deionised water, ERK1/2 were used in in vitro phosphorylation reactions.

### In vitro kinase activity assay

Immunoprecipitated ERK1/2 were incubated (20 min, 30 °C) with 5 μg of dephosphorylated MBP (Millipore, Temecula, CA, USA) in buffer containing 100 mM MOPS (pH 6.7 or 7.4), 0.5 mM EGTA, 1 mM DTT, 20 mM MgCl_2_, 200 μM ATP and EDTA-Free Protease and Phosphatase Inhibitor Tablets (Thermo Scientific, Rockford, IL) with the indicated concentration of freshly dissolved NaHCO_3_. Then, the proteins were denatured in SDS-PAGE sample loading buffer and subjected to immunoblotting with an anti-phospho-MBP antibody.

### Quantitative measurement of IL-6 levels

Measurement of the level of IL-6 in cell culture supernatants was carried out using an enzyme-linked immunosorbent assay (ELISA) kit (Cat # 430504, BioLegend, San Diego, CA) according to the manufacturer’s instructions.

### RNA extraction and RT-qPCR

RNA extraction with TRI reagent (Sigma–Aldrich) and reverse transcription using a RevertAid H Minus First-Strand cDNA Synthesis Kit (Thermo Fisher Scientific, K1631) were conducted according to the manufacturers’ protocols and described previous particulars [12]. PowerUp™ SYBR^®^ Green Master Mix (Thermo Fisher Scientific, A25742) and StepOnePlus Real-Time PCR System (Thermo Fisher Scientific) were used to perform qPCR with primers specific to *ACE2* (ACCAGTGGATGAAAAAGTGGTG and AGAAACATGGAACAGAGATGCG), *GAPDH* (GTCTCCTCTGACTTCAACAGCG and ACCACCCTGTTGCTGTAGCCAA) and *ACTB* (CACCATTGGCAATGAGCGGTTC and AGGTCTTTGCGGATGTCCACGT). The 2^−ΔΔCT^ method [13] was applied to calculate the relative level of *ACE2* mRNA.

### Statistical analysis

ImageJ software was employed for densitometric analysis of the immunoblotting bands. The expression levels of total kinases and GAPDH and Ponceau S staining were used for data normalisation. For relative quantification, the value of the mock treatment group was considered 1. The means ± standard deviations (SD) are shown. The significance of differences was calculated using a two-tailed *T*-test (Fig. S3a) or one-way or factorial ANOVA followed by Tukey’s post hoc test (other Figs). The means ± SDs of three independent experiments are presented. *, ** and *** indicate significant differences in ERK1/2 activity (*p* < 0.05, *p* < 0.01 and *p* < 0.001, respectively).

## Results

We focused on ECs (cell line EA.hy926) to test hypotheses regarding the potential role of CO_2_ in regulating ERK1/2 activity, because ECs in the lungs, heart and brain are strongly affected in COVID-19, and COVID-19 is considered a form of inflammatory endothelialitis [14, 15].

### ERK1/2 are activated by CO_2_ in vitro and in human cells

**Fig. 1:**
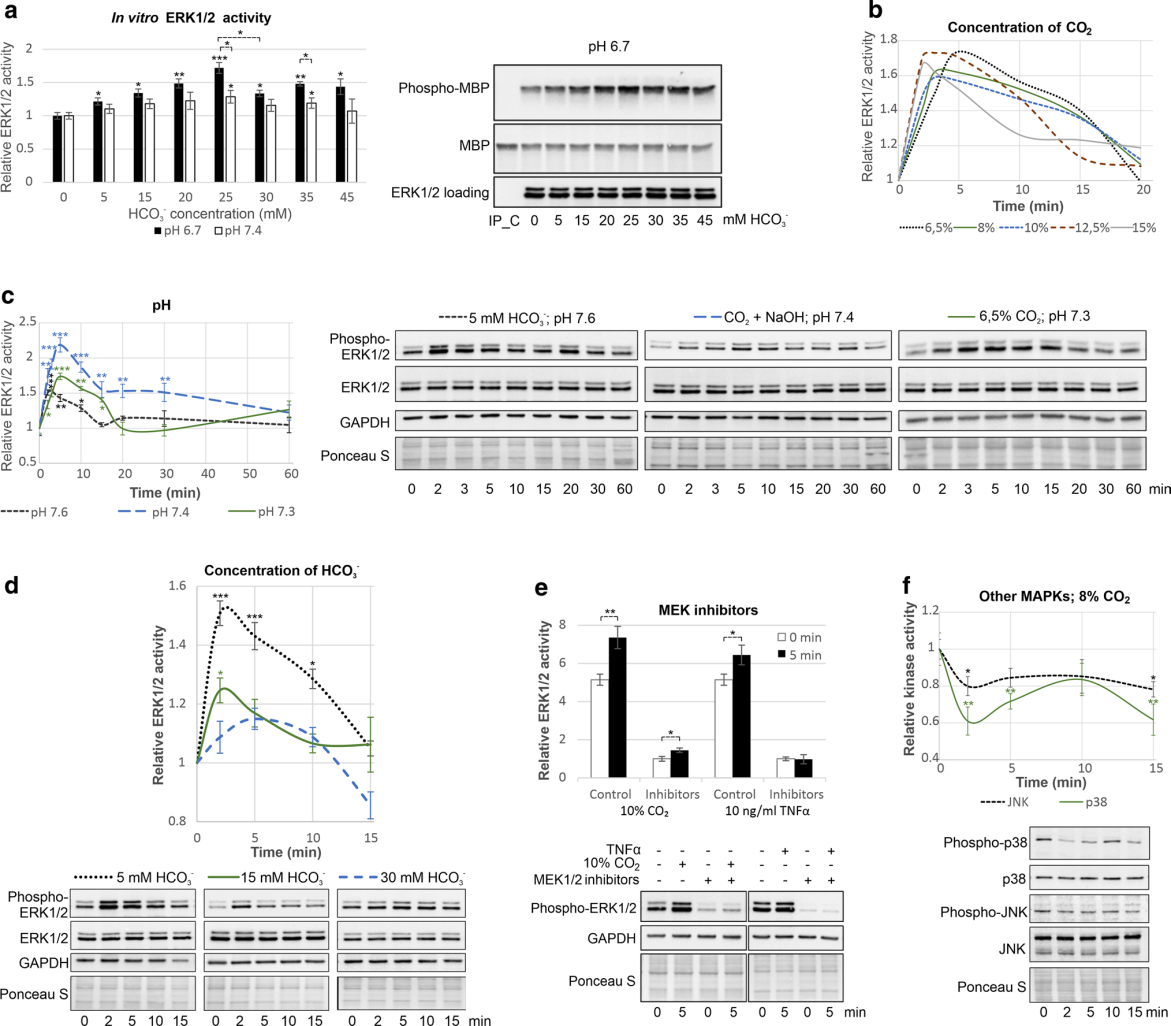
MAPKs are regulated by CO_2_. **a** CO_2_ directly regulates ERK1/2 activity in vitro. ERK1/2 immunoprecipitated from ECs was dephosphorylated and subjected to an in vitro kinase activity assay in the presence of the indicated concentration of HCO_3_^−^. Phosphorylation of MBP, which was used as an ERK1/2 substrate for in vitro phosphorylation reactions, was measured by immunoblotting with an anti-phospho-MBP antibody. The levels of ERK1/2 were determined using immunoblotting with an anti-ERK1/2 antibody, and MBP was visualised by Ponceau S staining. An immunoprecipitation control (IP_C), i.e. Dynabeads Protein G incubated with cell lysates without the anti-ERK1/2 antibody, was used to show the specificity of the assay. **b** In ECs, ERK1/2 were transiently activated by CO_2_ at a wide range of concentrations. Graphs for the individual CO_2_ concentrations with bars representing the SD are presented in Fig. S1a. **c** CO_2_ promotes ERK1/2 activation regardless of pH. Time courses of ERK1/2 activation at pH 7.4 (CO_2_ neutralised by NaOH) and at a lower (6.5% CO_2_) or higher (NaHCO_3_) pH are shown. **d** Time courses of HCO_3_^−^-induced ERK1/2 activation in ECs. **e** ERK1/2 were activated by CO_2_ independently of upstream MEK1/2 in ECs, as MEK1/2 inhibitors (2.5 µM U0126 and 25 µM PD98059) did not abolish the CO_2_-induced ERK1/2 activation but did prevent ERK1/2 activation by 10 ng/ml TNFα. In **b-e**, ERK1/2 activity was measured by immunoblotting with an anti-phospho-ERK1/2 antibody. Protein loading was assessed by both Ponceau S staining and immunoblotting with anti-GAPDH and anti-ERK1/2 antibodies. **f** JNK and p38 were inactivated by CO_2_ in ECs. JNK and p38 activity was determined by immunoblotting with anti-phospho-JNK and anti-phospho-p38 antibodies, respectively. The results of three independent experiments are presented

First, we tested whether ERK1/2, the most closely related human MAPKs to plant MAPKs, are directly regulated by CO_2_ (Fig. 1a). Inactive ERK1/2 immunoprecipitated from ECs were activated by HCO_3_^−^ in vitro in a dose-dependent manner up to 25 mM HCO_3_^−^ at pH 6.7 (32.4% CO_2_ and 67.6% HCO_3_^−^ in the reaction mixture), whereas activation was weak at pH 7.4 (8.7% CO_2_ and 91.2% HCO_3_^−^). This result indicates that dissolved CO_2_, but not HCO_3_^−^, activates ERK1/2. The decrease in ERK1/2 activity in the presence of 30 mM HCO_3_^−^, which is close to the concentration in plasma, suggests that ERK1/2 can be activated by both increasing and decreasing the cellular CO_2_ concentration. Then, we found that transient activation of ERK1/2 in ECs occurred at all tested CO_2_ concentrations (6.5–15%; Fig. 1b, S1a). It was clear that the higher the CO_2_ concentration was, the earlier and shorter the maximal ERK1/2 activation. Since CO_2_ causes acidification of solutions, we showed that the activation of ERK1/2 in ECs was also induced by both NaHCO_3_ (which increases the pH) and neutralised (pH 7.4) CO_2_ (Fig. 1c). Thus, ERK1/2 activation was triggered by CO_2_, not at a certain pH. Lower CO_2_-induced ERK1/2 activation was found at a higher pH (Fig. 1d), which further confirms the free CO_2_ is better able to activate MAPKs than HCO_3_^−^, as found in vitro and in plants [9]. To define whether CO_2_-dependent regulation of ERK1/2 activity is a universal process that takes place in different cell types, we confirmed that 10% CO_2_ induced transient ERK1/2 activation in BEAS-2B cells (Fig. S1b). To verify that CO_2_ directly activated ERK1/2 in ECs, we showed that ERK1/2 were still activated by CO_2_ in the presence of MEK1/2 inhibitors (U0126 and PD98059), while activation of ERK1/2 by TNFα was abolished under these conditions (Fig. 1e).

### CO_2_ inhibits active ERK1/2

Since p38 and JNK are inactivated by CO_2_ (Fig. 1f) and active plant MAPKs can be inactivated by HCO_3_^−^/CO_2_ in vitro [9], we asked whether human ERK1/2 are subject to such unusual conditional positive or negative regulation in ECs. To this end, ECs were exposed to 100 μM H_2_O_2_ because unlike most factors, which transiently activate ERK1/2 (typically up to 30 min), H_2_O_2_ at a concentration of 100 μM permanently activates ERK1/2. Activated ERK1/2 were successfully inhibited by 30 mM HCO_3_^−^ over a time range of 2 to 4 h (Fig. S2a). More stable ERK1/2 inactivation can be achieved by repeated treatment with buffered (pH 7.4) HCO_3_^−^ (Fig. S2b) or CO_2_ (Fig. S2c). Transient hypercapnia, or an increased pCO_2_, is a physiological condition affecting the blood vessels, and further experiments were conducted using a transient increase in the CO_2_ concentration to avoid the introduction of additional compounds (NaOH, HEPES) to the cell culture medium.

**Fig. 2:**
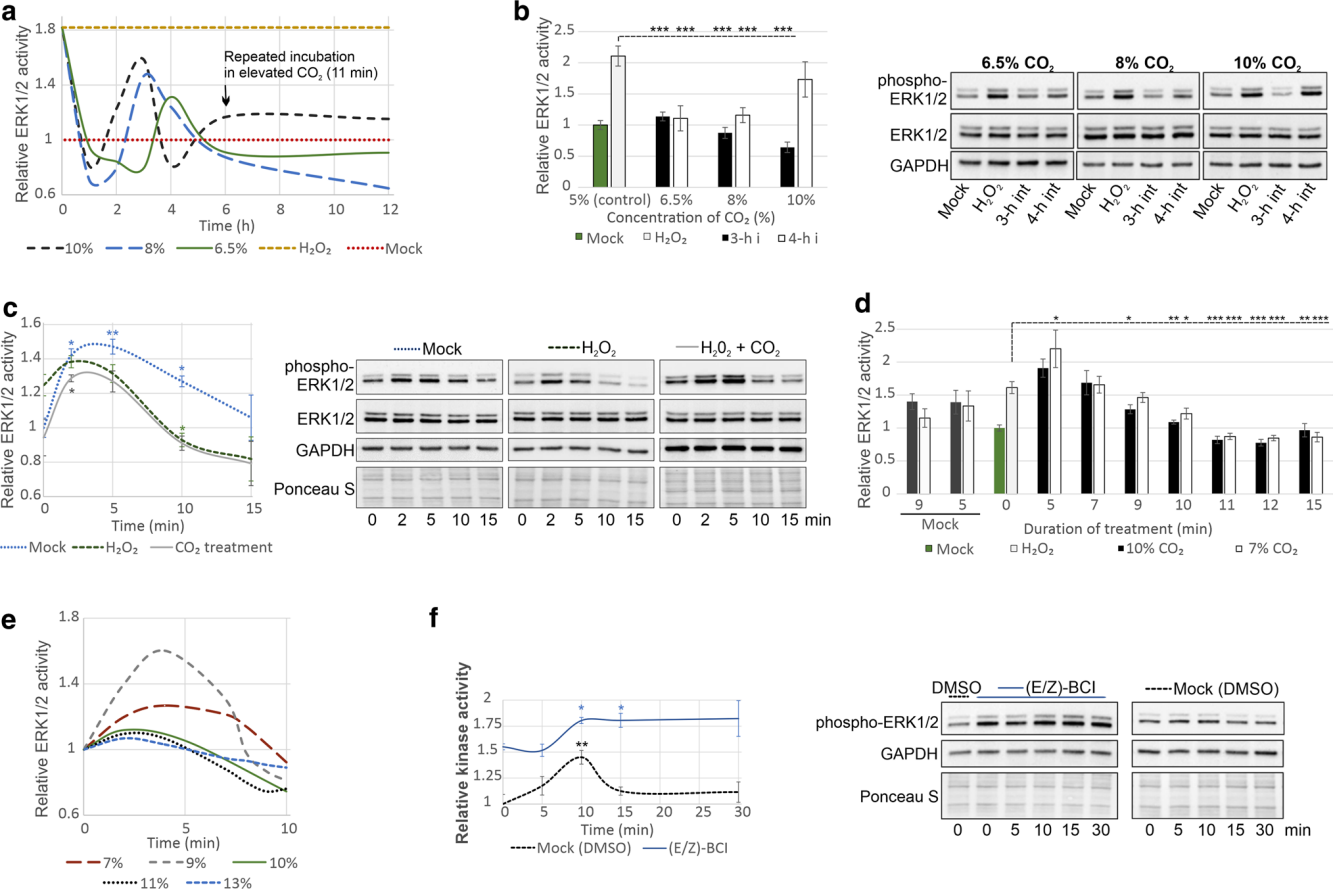
Active MAPKs are inhibited by CO_2_. In **a**-**e**, ERK1/2 were activated by treatment of ECs with 100 μM H_2_O_2_ for 12 h before inactivation by CO_2_. **a** CO_2_ inhibited the activity of ERK1/2 preactivated by 100 μM H_2_O_2_. Additional incubation with elevated CO_2_ (6 h after the first incubation) promoted stable ERK1/2 inactivation. Graphs showing the time course of ERK1/2 activity in response to particular CO_2_ concentrations with bars representing the SD are depicted in Fig. S2d. **b** A stable decrease in ERK1/2 activity was achieved by retreatment with elevated CO_2_. Additional incubations were initiated at different phases of ERK1/2 re-activation in **a**, i.e. in the third and fourth hour. **c** ERK1/2 inactivation by CO_2_ was preceded by short activation. Inactivation of ERK1/2 preactivated with 100 μM H_2_O_2_ or additional treatment with 10% CO_2_ (11 min, three times at 3-h intervals) was earlier than that of control ERK1/2. **d** Exposure to elevated CO_2_ for a sufficient duration was crucial for ERK1/2 inactivation. ECs in which ERK1/2 was activated by 100 μM H_2_O_2_ were incubated for the indicated times in 7 or 10% CO_2_ (three treatments at 3-h intervals). ERK1/2 activity measured 2 h after the last treatment is shown. **e** Timing of ERK1/2 inactivation in response to different CO_2_ concentrations. Graphs for the individual CO_2_ concentrations with bars representing the SD are shown in Fig. S2e. **f** ERK1/2 inactivation by CO_2_ was MKP-1-dependent. Time course of ERK1/2 activity in ECs treated with 10% CO_2_ in the presence of either DMSO or BCI, a specific MKP-1 inhibitor. In **a**–**f**, ERK1/2 activity was assessed by immunoblotting with an anti-phospho-ERK1/2 antibody and normalised to protein loading, which was assessed by Ponceau S staining and immunoblotting with anti-GAPDH and anti-ERK1/2 antibodies. The results are presented as the mean ± SD of three independent experiments

Efficient inhibition of ERK1/2 below baseline levels was induced by an increase in the CO_2_ concentration for 11 min (Figs. 2a, S2d). After a strong decline, ERK1/2 activity was partially and temporarily restored starting at the 2nd or 3rd h depending on the CO_2_ concentration. The decrease in ERK1/2 activity could be prolonged by additional incubations with CO_2_ at an elevated concentration. Additional incubations were initiated at different phases of ERK1/2 re-activation (Fig. 2b), and the most efficient ERK1/2 inactivation was observed when incubation was performed during peak ERK1/2 activity. In contrast, statistically insignificant ERK1/2 inactivation was noticed when CO_2_ was applied again when ERK1/2 was mostly inactive (4th h, 10% CO_2_).

Effective inactivation of ERK1/2 was noted for different CO_2_ concentrations. Therefore, we looked for other factors affecting ERK1/2 activity induced by CO_2_. After stimulation with H_2_O_2_ alone or in combination with 10% CO_2_ (11 min, 3 times, 3-h intervals), ERK1/2 were initially activated by CO_2_ before inactivation, and the time required to achieve baseline ERK1/2 activity was much shorter in H_2_O_2_-treated ECs than in control ECs (less than 10 and 15 min, respectively; Fig. 2c). Therefore, we asked whether the duration of incubation with elevated CO_2_ is essential for the effect of ERK1/2 inhibition. To this end, we analysed the inhibition of H_2_O_2_-induced ERK1/2 following three incubations with elevated (7 or 10%) CO_2_ for different duration at 3-h intervals (Fig. 2d). We found that incubations longer than 7 min were required to reduce ERK1/2 activity and that incubations longer than 9 min decreased ERK1/2 activity below the baseline level. Overall, the longer the incubation periods with elevated CO_2_, the more effective the ERK1/2 inhibition. However, exposure to elevated CO_2_ for longer than 12 min did not enhance the inhibitory effect of CO_2,_ and incubation for shorter than 7 min enhanced ERK1/2 activity. We also examined the response of ERK1/2 preactivated by H_2_O_2_ to different levels of CO_2_ (Figs. 2e, S2e). At a concentration of 7–9%, CO_2_ first triggered marked ERK1/2 activation followed by inactivation after 7 min, whereas the inactivation caused by 10–13% CO_2_ occurred earlier and was preceded by only weak ERK1/2 activation.

The kinetics of CO_2_-induced ERK1/2 activation followed by inactivation resembles the pattern of ERK1/2 activity changes evoked by dex (Fig. S2f). Since ERK1/2 inactivation by dex depends on increased MKP-1 expression [16], we checked whether this is also the case for CO_2_. Indeed, ERK1/2 were not inactivated in the presence of BCI, a specific MKP-1 inhibitor (Fig. 2f).

### ERK1/2 contribute to the response to RBD in ECs

**Fig. 3:**
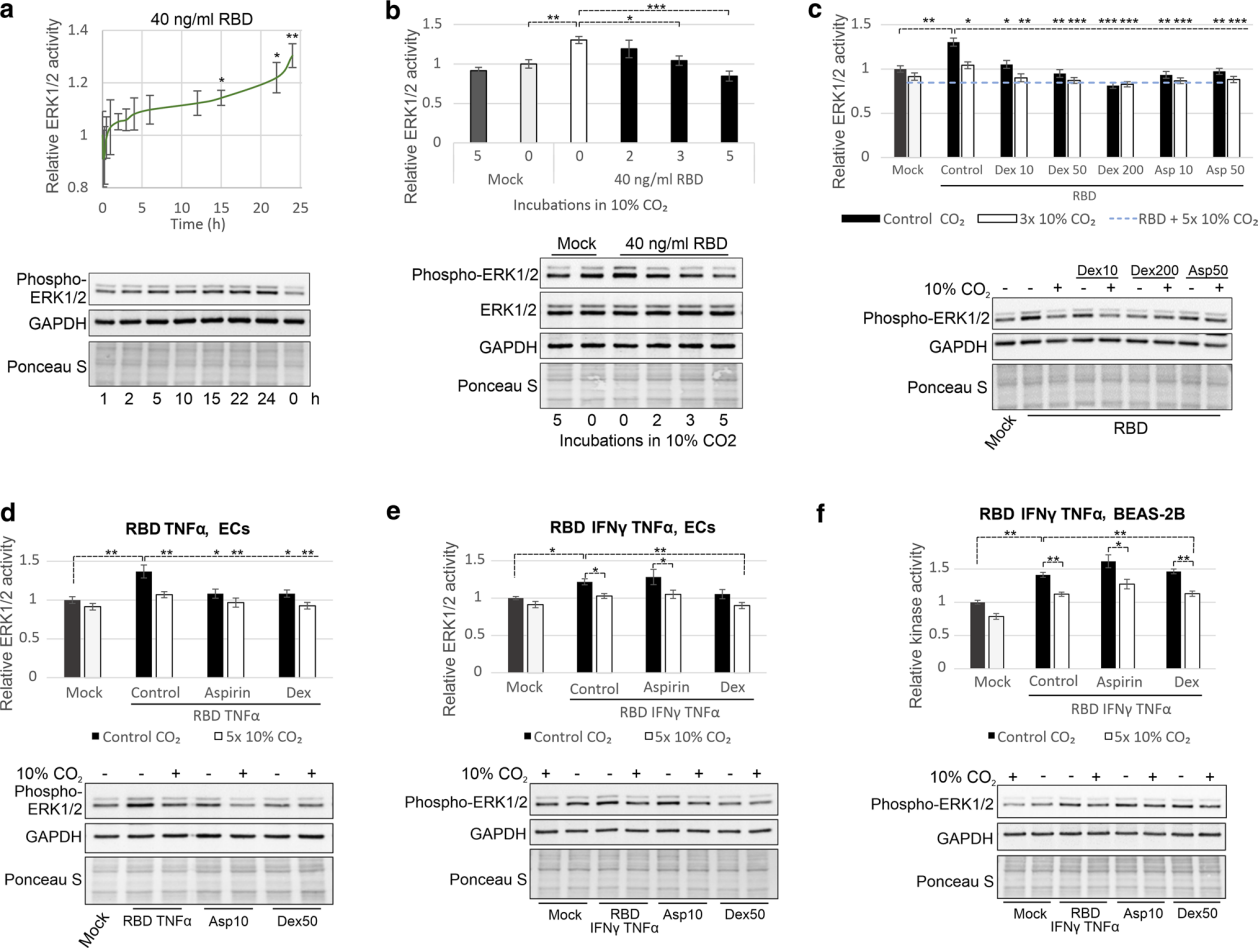
RBD-induced ERK1/2 activation can be inhibited by CO_2_. **a** Time course of ERK1/2 activation by 40 ng/ml RBD in ECs. **b** CO_2_ decreased ERK1/2 activity enhanced by 40 ng/ml RBD in ECs. The indicated number of treatments with 10% CO_2_ (11 min, a 3-h interval for “3 × CO_2_” and “5 × CO_2_” and a 6-h interval for “2 × CO_2_”) was applied 2 h before collection of ECs and 22 h after RBD administration. **c** Comparison of the efficiency of ERK1/2 activity inhibition by dex (10, 50 or 200 nM), 10 or 50 μM aspirin (asp) and 10% CO_2_. ECs were incubated with 40 ng/ml RBD for 16 h. Then, dex and aspirin were applied directly after the first treatment with 10% CO_2_ (11 min) 8 h before collection of ECs. **d–f** Efficacy of ERK1/2 inhibition by 10% CO_2_ (5 treatments, 12 min, 3-h interval), 10 μM aspirin and 50 nM dex. ECs (**d–e**) or BEAS-2B cells (**f**) were treated with 40 ng/ml RBD, 10 ng/ml TNFα and/or 7.5 ng/ml IFNγ as indicated for 10 h. Then, specified treatments were applied, and the cells were collected 14 h later. ERK1/2 activity was determined by immunoblotting with an anti-phospho-ERK1/2 antibody, and protein loading was assessed by Ponceau S staining and immunoblotting with anti-GAPDH and anti-ERK1/2 antibodies. The means ± SDs of three independent experiments are presented

In light of the current COVID-19 pandemic, we asked whether elevated CO_2_ could be effective in inhibiting the cytokine production caused by SARS-CoV-2 infection. It is accepted that the ACE2 receptor is expressed in ECs [14], and we showed that the expression of ACE2 was increased by RBD in EA.hy926 cells at both the protein and mRNA levels (Fig. S3a). SARS-CoV-2 is able to infect human blood vessel organoids and ECs in vitro without undergoing viral replication and induces a clear proinflammatory response [17]. Therefore, we were able to demonstrate that in ECs, ERK1/2 activity in response to 40 ng/ml RBD increased gradually up to ~ 30% after 24 h (Fig. 3a, Fig. S3b), according to data from epithelial cells [18, 19].

As diabetes mellitus is one of the most significant risk factors for severe COVID-19, we compared ERK1/2 activity in response to RBD in ECs exposed to 5.55 and 25 mM glucose (Fig. S3c). Fifteen hours after RBD application, ERK1/2 were activated in a concentration-dependent manner up to 400 ng/ml RBD, and ERK1/2 activation was higher in ECs exposed to 25 mM glucose than to those exposed to 5.55 mM glucose. This is consistent with the glucose-mediated induction of ACE2 expression, SARS-CoV-2 load, and cytokine and ROS production [20, 21]. Therefore, all further data were obtained from ECs cultured in 25 mM glucose.

### RBD-activated ERK1/2 are inhibited by CO_2_

Although activation of ERK1/2 in response to RBD was weaker than that in response to H_2_O_2_, RBD-induced ERK1/2 activation was less effectively inhibited, and exposure to 10% CO_2_ three times at an interval of 3 h was necessary to retain baseline ERK1/2 activity (Fig. 3b). To directly compare the therapeutic effect of CO_2_ with that of anti-inflammatory drugs that inhibit ERK1/2 activity, we applied 10, 50 or 200 nM dex and 10 or 50 μM aspirin. Exposure to 10% CO_2_ five times for 12 min more efficiently inhibited ERK1/2 activity than aspirin and 10 or 50 nM dex (Fig. 3c). Importantly, CO_2_ acted synergistically with dex and aspirin, except dex was used at a concentration of 200 nM. Interestingly, the aspirin-induced changes in ERK1/2 activity over time were distinct from those of dex and CO_2_; ERK1/2 were inactivated beginning from the 5th min but did not exhibit initial strong activation (Fig. S3d).

Then, we aimed to show the versatility of CO_2_-induced inactivation of ERK1/2. In contrast to studies showing that ACE2 is not expressed at the mRNA level in BEAS-2B cells [22], we confirmed the results of other groups [19] demonstrating the presence of ACE2 protein and weak transient activation of ERK1/2 by RBD in BEAS-2B cells (Fig. S4a-b). However, RBD-induced ERK1/2 activation was suppressed after 3 h, consistent with the known decrease in ACE2 expression induced by spike in epithelial cells [8]. Therefore, we measured ERK1/2 activity after induction by RBD in combination with IFNγ and TNFα since IFNγ and TNFα are responsible for acute SARS-CoV-2 infection along with IL-6 [23] and activate ERK1/2 in many cell types to promote IL-6 production. IFNγ was found to play a key role in potent and long-term ERK1/2 activation in BEAS-2B cells (Fig. S4a, b). Conversely, ACE2 expression was decreased at the same time (Fig. S4a), supporting its role as an ERK1/2 inhibitor [6, 7].

Unlike in BEAS-2B cells, TNFα enhanced RBD-induced ERK1/2 activation in ECs (Fig. S4c-e). Therefore, we applied RBD in together with TNFα or RBD together with TNFα and IFNγ to induce ERK1/2 activity in ECs and BEAS-2B cells (Fig. 3d–f). Following five 12-min incubations in 10% CO_2_, ERK1/2 activity was effectively blocked under these conditions in both cell lines (Fig. 3d–f), while aspirin failed to inhibit ERK1/2 activity in the presence of IFNγ in ECs (Fig. 3e). Neither aspirin nor dex was able to block the activity of ERK1/2 in BEAS-2B cells (Fig. 3f). Importantly, CO_2_ enhanced the decline in ERK1/2 activity in the presence of dex and aspirin in all tested combinations, both in BEAS-2B cells and ECs (Fig. 3d–f).

Notably, aspirin is known to induce aspirin-exacerbated respiratory disease (AERD), in which IFNγ plays a crucial role [24]. Moreover, aspirin has also been reported to induce ERK1/2 activity [25]. The inability of dex to efficiently inhibit ERK1/2 (Fig. 3f) may result from the synergistic induction of Toll-like receptor 2 (TLR2; an indirect activator of ERK1/2) by dex, TNFα, and IFNγ in human respiratory epithelial cells, including BEAS-2B cells [26]. In addition, dex is only effective in cells, tissues and organs with active glucocorticoid receptors and signalling machineries and ERK1/2 activity plays a role in the development of corticosteroid resistance [27].

**Fig. 4:**
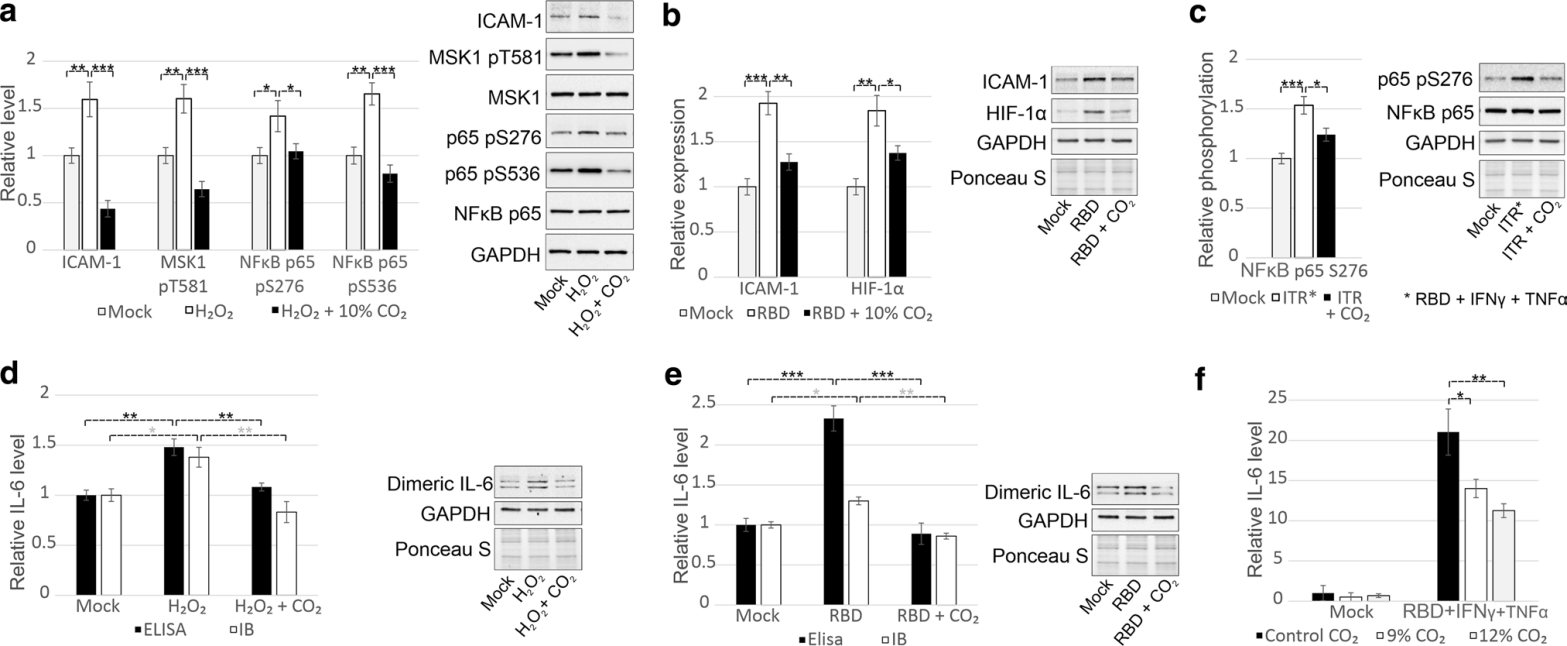
ERK1/2-dependent downstream signalling and cellular responses are regulated by CO_2_ in ECs. **a** Increased expression of ICAM-1 and phosphorylation of MSK1 at T581 and NFkB p65 at S276 and S536 in response to 100 μM H_2_O_2_ were abrogated by treatment with 10% CO_2_ (11 min, 4 times, 3-h interval). **b** The expression of HIF-1α and ICAM-1 were induced by 40 ng/ml RBD and could be decreased by 10% CO_2_ in ECs (4 incubations, 11 min, 3-h interval). **c** The phosphorylation of NFkB p65 at S276 was increased in response to RBD, TNFα and IFNγ combination and could be decreased by 10% CO_2_ (4 incubations, 11 min, 3-h interval) in ECs. **d–e** H_2_O_2_-induced (**d**) and RBD-triggered (**e**) production of IL-6 by ECs was diminished by 10% CO_2_ (11 min, 3-h interval). The level of IL-6 secreted into the cell culture supernatant was determined by ELISA. Dimeric intracellular IL-6 was visualised by immunoblotting with an anti-IL-6 antibody, and the expression of this form of IL-6 was normalised to the signal obtained with an anti-GAPDH antibody. **f** CO_2_ at a concentration of 9–12% inhibited the production of IL-6 which was induced by RBD, TNFα and IFNγ combination. IL-6 production was measured by ELISA. The results of three biological replications are presented as the mean ± SD

Next, we traced MAPK-dependent proteins that lead to proinflammatory responses. We showed that the expression of hypoxia-inducible factor 1 alpha (HIF-1α) and intercellular adhesion molecule 1 (ICAM-1) and phosphorylation of NFκB p65 and mitogen- and stress-activated protein kinase 1 (MSK1) were induced by H_2_O_2_ (Fig. 4a) or RBD alone (Fig. 4b) or RBD together with TNFα and IFNγ (Fig. 4c) and could be decreased by CO_2_ in ECs. Since tocilizumab, an antibody against the IL-6 receptor, is one of the most effective drugs for patients with severe COVID-19, we also demonstrated that the levels of both secreted and dimeric intracellular IL-6 were increased in response to both RBD and H_2_O_2_ and decreased by CO_2_ (Fig. 4d–f).

**Fig. 5:**
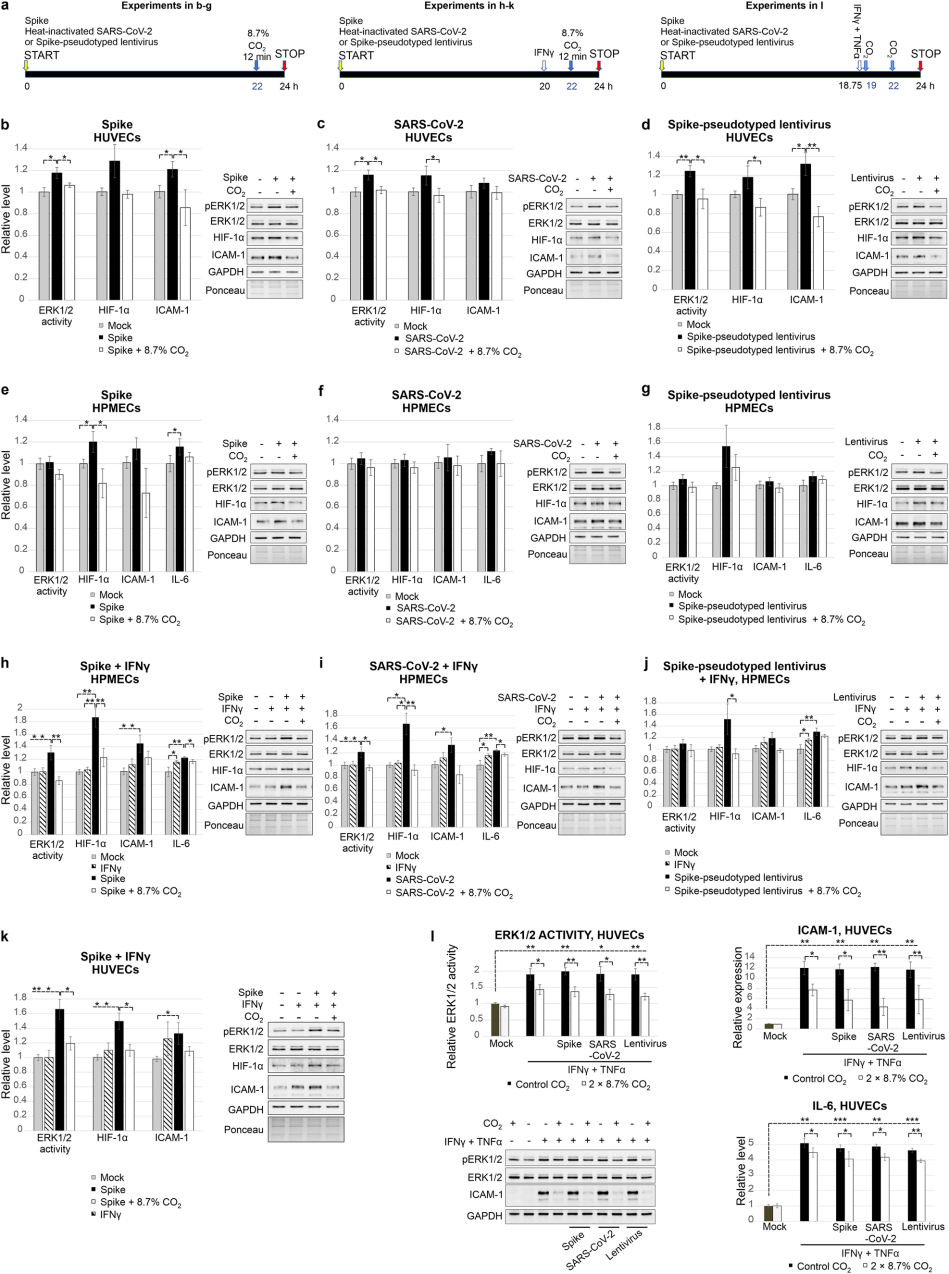
Responses to SARS-CoV-2 components and treatment with CO_2_ in primary ECs. ERK1/2 activity was determined by immunoblotting with an anti-phospho-ERK1/2 antibody, and protein loading was assessed by Ponceau S staining and immunoblotting with anti-GAPDH and anti-ERK1/2 antibodies. Changes in the expression of specific proteins were detected with anti-HIF-1α and anti-ICAM-1 antibodies. IL-6 level was measured by ELISA. The means ± SDs of three independent experiments are presented. **a** The scheme of the course of experiments. In **b–g**, ECs were incubated with mock or the indicated SARS-CoV-2 derivative for 22 h. Then, 8.7% CO_2_ was applied for 12 min. Cells were frozen 24 h after the start of the experiment. In **h**–**l**, ECs were incubated with mock or the indicated SARS-CoV-2 derivative for 20 h. Then, 7.5 ng/ml IFNγ was added. After 2 h, treatment with 8.7% CO_2_ for 12 min was conducted. Cells were collected 24 h after the beginning of the experiment. **b** 50 ng/ml spike, HUVECs. **c** Heat-inactivated SARS-CoV-2, USA-WA1/2020 isolate, HUVECs. A number of administered viral particles corresponded to the multiplicity of infection (MOI) of 7.5. **d** Spike-pseudotyped lentivirus, HUVECs. **e** 50 ng/ml spike, HPMECs. **f** Heat-inactivated SARS-CoV-2, USA/CA_CDC_5574/2020 isolate, MOI 3, HPMECs. **g** Spike^D614G^-pseudotyped lentivirus, HPMECs. **h** 50 ng/ml spike with 7.5 ng/ml IFNγ, HPMECs. **i** Heat-inactivated SARS-CoV-2, USA/CA_CDC_5574/2020 isolate with 7.5 ng/ml IFNγ, HPMECs. **j** Spike^D614G^-pseudotyped lentivirus with 7.5 ng/ml IFNγ in HPMECs. **k** 50 ng/ml spike with 7.5 ng/ml IFNγ, HUVECs. **l** HUVECs were incubated with mock or the indicated SARS-CoV-2 derivative for 18 h 45 min. Then, 7.5 ng/ml IFNγ and 10 ng/ml TNFα were added. After 15 min and subsequently after 3 h 15 min, the cells were treated with 8.7% CO_2_ for 12 min. Cells were collected 24 h after the beginning of the experiment. pERK1/2—phosphorylated ERK1/2

To confirm the impairment of SARS-CoV-2-induced inflammation by CO_2_ in a more natural context, we employed primary ECs from the umbilical vein and from the pulmonary microvasculature, trimeric spike glycoprotein, spike-pseudotyped lentivirus and heat-inactivated SARS-CoV-2. At a low glucose concentration (5.55 mM), ERK1/2 activation by spike, spike-pseudotyped lentivirus or heat-inactivated SARS-CoV-2 was clearly stronger in HUVECs (Fig. 5a–d) than RBD-induced ERK1/2 activation in the EA.hy926 cell line (Fig. S3c). However, the change in ERK1/2 activity in HPMECs exposed to spike, spike-pseudotyped lentivirus or heat-inactivated SARS-CoV-2 (Fig. 5e–g) did not reach statistical significance similar to RBD-induced activation of ERK1/2 in EA.hy926 cells under low glucose conditions. The lower activation of ERK1/2 in HPMECs compared to HUVECs may result from conducting experiments on dividing HPMECs (with lower cell density). This approach was necessary to maintain the purity of the culture because the microvasculature EC preparations are usually contaminated with other cell types and the amount of contaminants increases with the duration of the culture. In addition, HPMECs were grown on plasma fibronectin-coated vessels, and plasma fibronectin is known to modulate a number of cellular responses [28]. Then, we evaluated the activation of ERK1/2 in HPMECs in the presence of IFNγ. Whereas IFNγ alone did not activate ERK1/2, spike with IFNγ (Fig. 5h) or heat-inactivated SARS-CoV-2 accompanied by IFNγ (Fig. 5i) efficiently activated ERK1/2 in 5.55 mM glucose. A less pronounced effect was induced by spike-pseudotyped lentivirus in the presence of IFNγ (Fig. 5j). SARS-CoV-2-derivative IFNγ synergism was not limited to HPMECs, as spike-induced ERK1/2 activation in HUVECs was enhanced (1.74-fold) on the addition of IFNγ. (Fig. 5k).

Primary ECs are very responsive to treatment with CO_2_. Only one treatment with CO_2_ (8.7%, 12 min) efficiently inhibited not only ERK1/2 activity but also the increase in IL-6, ICAM-1 and HIF-1α expression in primary ECs (Fig. 5b–k). IFNγ stimulated an ERK1/2-independent increase in the IL-6 expression level in HPMECs. Thus, CO_2_ repressed IL-6 expression co-induced by IFNγ and SARS-CoV-2 derivatives to the levels following IFNγ induction, in contrast with ICAM-1 and HIF-1α expression levels, which were repressed to near control levels. Very high induction of ICAM-1 and IL-6 expression on synergistic action of TNFα and IFNγ (also in combination with the presence of spike, heat-inactivated SARS-CoV-2 or spike-pseudotyped lentivirus) can be partially repressed by two treatments with CO_2_ after TNFα and IFNγ administration (Fig. 5l).

Overall, the current work revealed that CO_2_ is a potent regulator of MAPK activity. The effect of its action can be tailored by adjusting the concentration and duration of the treatment.

## Discussion

In the present work, we identified a novel regulatory mechanism and cross-talk between fundamental signalling pathways through the regulation of ERK1/2 by CO_2_. Its regulatory effect on the inflammatory response presented in this paper is only a one example of the possible applications of CO_2_, and our findings have much broader significance due to involvement of MAPKs in many developmental processes and oncogenesis. Accordingly, a number of MAPK inhibitors have been approved as potent drugs for the treatment of numerous diseases, including cancers. However, they cannot be fully exploited due to their toxicity. CO_2_, which is safe under controlled conditions, could overcome these limitations. The elucidation of the molecular basis of the action of CO_2_ in this paper may be the first step in broadening the use of CO_2_ in clinical practice, as there are many preclinical studies showing the effects of CO_2_, including its anti-tumour activity [29].

Despite the obvious detrimental effects of long-term exposure to elevated CO_2_ concentrations [30], short-term inhalation of 5–8% CO_2_ as a treatment for inflammation appears to be unprecedentedly safe because CO_2_ is a natural compound that constantly affects the body, has transient effects and is easily removed via the lungs and kidneys. The amount of CO_2_ supplied by a single administration of 5–8% CO_2_ is equivalent to that produced by breath holding several times for less than half a min. However, unlike breath holding, oxygen is also delivered to the lungs at the same time.

Hundreds of clinical trials have confirmed that short-term inhalation of 5% CO_2_ is absolutely safe and that CO_2_ does not have systemic effects when inhaled at a concentration of up to 8% [31]. Inhalation of 5% CO_2_ is used in magnetic resonance imaging due the potent vasodilatory ability of CO_2_. In psychiatry, inhalation of up to 35% CO_2_ is applied to induce a panic attack, and it has been postulated that studies should be conducted with a minimum of 7.5% CO_2_ since 5% CO_2_ causes physiological and psychological effects that are too weak [32]. On the other hand, in healthy subjects, 10% CO_2_ (i.e. a very high concentration) increases pulmonary artery pressure. However, inhaled 5% CO_2_ reduces existing pulmonary hypertension [33]. Correspondingly, in this work, we found that CO_2_ activates inactive ERK1/2 and inactivates active ERK1/2.

Inhaled CO_2_ is effective for the treatment of febrile seizures [34], central sleep apnoea syndrome [35], and seizures in epilepsy patients [36] and counteracts central retinal artery occlusion [31]. CO_2_ enables oxygen to be released from haemoglobin [37]. As a result of inhalation of 2–5% CO_2_, the oxygenation of the arterial blood increases, which results in better oxidation and susceptibility of tumours to radiotherapy [38]. Similarly, in infants ventilated mechanically after the Norwood procedure, administration of CO_2_ increases blood oxygen saturation and the supply of oxygen to tissues [39]. CO_2_ is not only increasingly being used for carboxytherapy in aesthetic medicine but also increasingly being administered via inhalation and baths for the treatment of kidney, cardiovascular and neurological diseases.

Although CO_2_ sensing seems to be a universal process among eukaryotes, currently recognised HCO_3_^−^ sensors, namely, GCs and ACs, are absent in many eukaryotes. Moreover, CO_2_ signalling pathways, which are crucial for vertebrates, are independent of GCs and ACs, including the pathways responsible for the regulation of breathing in response to changes in CO_2_ concentrations [40], CO_2_ sensing by olfactory sensory neurons [3], and HCO_3_^−^-dependent regulation of spontaneous heart rate and cardiac force development. Interestingly, the activity of AC and GC in *Dictyostelium discoideum* depends on DdERK2, the activation of which is independent of MEKs [41]. Yeast proteins involved in CO_2_ signalling are functionally closely associated with MAPKs; Sch9 kinase [42] is regulated by Hog1 MAPK, and Ptc2 is a MAPK phosphatase that dephosphorylates Hog1 [43]. Intriguingly, ERK1/2, JNK and p38 are clearly involved in carbon monoxide signalling [44].

In this work, we focused on long-term activation of ERK1/2 to clearly demonstrate the effectiveness of CO_2_ at the level of MAPK activity. However, activation of MAPKs in response to many factors is transient. Regarding this type of MAPK activation, the effectiveness of both MAPK inhibitors [45] and a single 10-min administration of 5% CO_2_ [46] in vivo in preventing LPS-induced development of pneumonia has already been proven.

The conclusions from the present work are consistent with previous studies; CO_2_-induced activation of inactive ERK1/2 and inactivation of active ERK1/2 have been demonstrated in pheochromocytoma cells [47]. However, these results were not interpreted to be the direct influence of 20% CO_2_ on ERK1/2 activity. More recently, Xu et al. [48] showed that ERK1/2 is inactivated by CO_2_ in rat skeletal muscle.

Although ERK1/2, JNK and p38 have been shown to facilitate the replication of many viruses and nuclear export of viral ribonucleoprotein complexes, the MAPK pathway that is currently attracting the most attention is the pathway involved in the regulation of proinflammatory cytokine expression. Dex is widely used in the treatment of inflammation in severe COVID-19. However, the serious conditions of patients often result from comorbidities, and contraindications to the use of dex coincide with factors that increase the risk of a severe COVID-19. In addition, among other factors, chronic obstructive pulmonary disease (COPD), cigarette smoke extract, bleomycin‐induced acute lung injury and oxidative stress have been shown to cause resistance to corticosteroids in bronchial and alveolar epithelial cells. We have shown that the inhibitory effect of lower doses of dex in combination with CO_2_ on ERK1/2 is as marked as that of a high dose of dex. Moreover, CO_2_ treatment is the most effective method for inactivating ERK1/2 in epithelial cells in the presence of increased IFNγ and TNFα levels, which is characteristic of severe COVID-19.

In addition to a cytokine storm, infection with SARS-CoV-2 induces ROS generation [20]. In severe COVID-19, ROS and cytokine production in the lungs is enhanced by hyperoxia (resulting from oxygen support) [49] and mechanical ventilation [50–52]. These processes exacerbate lung injury, which can be reduced by both CO_2_ treatment and inhibition of MAPKs [49]. CO_2_ clearly inhibits ROS generation in many human and mouse tissues and stimulates the production of antioxidants [53]. There are other overlapping effects of CO_2_ and MEK inhibitors. MAPK inhibitors [45] or 5% CO_2_ inhalation for 10 min [46] is protective against LPS-induced pneumonia in mice. CO_2_ [54] and MEK inhibitors [19] attenuate the release of proinflammatory cytokines. Similarly, both synthetic MAPK inhibitors [55] and CO_2_ [56] counteract reperfusion and oxidative brain injury after ischaemic stroke, which is a frequent complication following SARS-CoV-2 infection. Therefore, the total CO_2_ concentration is lower in deceased patients than in patients who have recovered from COVID-19 [57]. In addition, it has been observed that current smoking protects against the development of severe COVID-19 symptoms [58].

## Conclusions

We have shown that CO_2_ is a potent inhibitor of ERK1/2 activity that acts independently of cell type and proinflammatory cytokines, which is in contrast to cytokine-susceptible aspirin and dex. Unlike dex and aspirin, CO_2_, which readily penetrates tissues and cells and acts independently of appropriate transporters, receptors and signalling pathways, inactivates mainly overactivated ERK1/2. Thus, our study supports previous report [59]. We encourage researchers to undertake further studies on CO_2_ as a potential therapeutic agent for COVID-19.

## Supplementary Information

Below is the link to the electronic supplementary material.

Supplementary file1 (PDF 973 KB)

## Data Availability

All the data generated or analysed during this study are included in this published article and its supplementary information files. Raw images generated during the current study are available from the corresponding author on reasonable request.

## Abbreviations

AC: Adenylyl cyclase
ACE2: Angiotensin-converting enzyme 2
BCI: (E/Z)-BCI hydrochloride
CO_2_: Carbon dioxide
COVID-19: Coronavirus disease 2019
dex: Dexamethasone
DMSO: Dimethyl sulfoxide
ECs: Endothelial cells
EGM-2: Endothelial cell growth medium-2
EGM-2 MV: Microvascular endothelial cell growth medium-2
ELISA: Enzyme-linked immunosorbent assay
ERK1/2: Extracellular signal-regulated kinases 1 and 2
FastAP: Fast alkaline phosphatase
GAPDH: Glyceraldehyde 3-phosphate dehydrogenase
GC: Guanylyl cyclase
HIF-1α: Hypoxia-inducible factor 1 alpha
HPMECs: Human pulmonary microvascular endothelial cells
HUVECs: Human umbilical vein endothelial cells
ICAM-1: Intercellular adhesion molecule 1
IFNγ: Interferon-gamma
IL-6: Interleukin-6
IP_C: Immunoprecipitation control
JNK: C-Jun N-terminal kinase
LPS: Lipopolysaccharides
MAPK: Mitogen-activated protein kinase
MBP: Myelin basic protein
MEK: MAPK kinase
MKP: MAPK phosphatase
MSK1: Mitogen- and stress-activated protein kinase 1
NFκB: Nuclear factor kappa-light-chain-enhancer of activated B cells
PBS: Phosphate-buffered saline
PBST: PBS with 0.1% Tween-20
pCO_2_: Partial pressure of CO_2_
RBD: Receptor-binding domain of the SARS-CoV-2 spike protein
ROS: Reactive oxygen species
TNFα: Tumour necrosis factor-α
SD: Standard deviation
TCID_50_: Tissue culture infectious dose 50
