# Supplementary material for: Carbon dioxide inhibits COVID-19-type proinflammatory responses through extracellular signal-regulated kinases 1 and 2, novel carbon dioxide sensors
Source: Cell Mol Life Sci. 2021 Nov 6;78(24):8229–42. doi: 10.1007/s00018-021-04005-3 (PMC8571007; doi:10.1007/s00018-021-04005-3)
Supplement: Supplementary file 1 — Supplementary file1 (PDF 973 KB) [file 18_2021_4005_MOESM1_ESM.pdf]

Supplementary Information

Figures S1 – S4

Carbon dioxide inhibits COVID-19-type proinflammatory responses through extracellular signal-regulated kinases 1 and 2, novel carbon dioxide sensors

Cellular and Molecular Life Sciences

Hanna Galganska[a], Wieslawa Jarmuszkiewicz[b*] and Lukasz Galganski[b*]

[a] Molecular Biology Techniques Laboratory, Faculty of Biology, Adam Mickiewicz University, Uniwersytetu Poznanskiego 6, 61-614 Poznan, Poland

[b] Laboratory of Mitochondrial Biochemistry, Department of Bioenergetics, Faculty of Biology, Adam Mickiewicz University, Uniwersytetu Poznanskiego 6, 61-614 Poznan, Poland

[*] Correspondence to: wieslawa.jarmuszkiewicz@amu.edu.pl

[*] Correspondence to: lukasz.galganski@amu.edu.pl

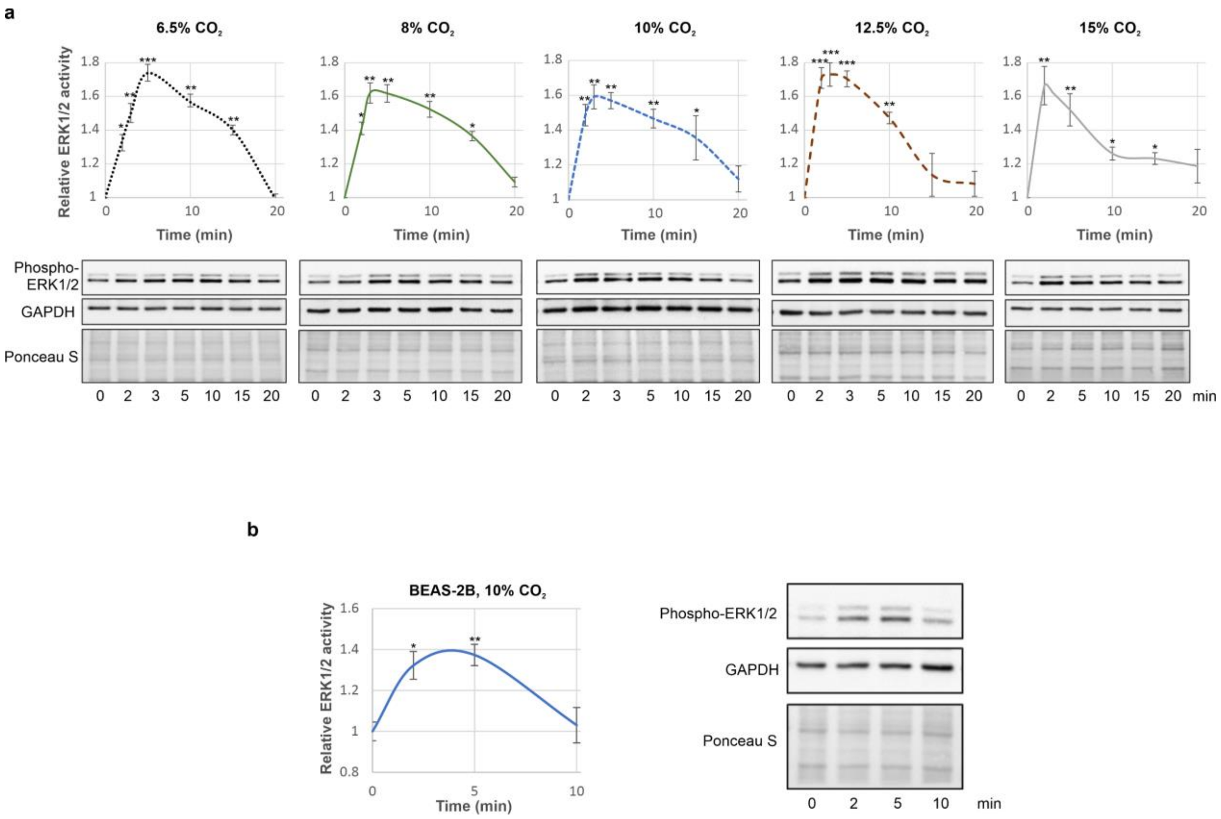

Fig. S1 Data supporting CO_2-induced ERK1/2 activation**. a** Transient activation of ERK1/2 by CO_2 at different concentrations in ECs. **b** Regulation of ERK1/2 activity over time in response to 10% CO_2 in BEAS-2B cells. ERK1/2 activity was evaluated using immunoblotting with an anti-phospho-ERK1/2 antibody, and protein loading was assessed using Ponceau S staining and immunoblotting with anti-GAPDH and anti-ERK1/2 antibodies. The results of three independent experiments are presented as the mean ± SD. *, ** and *** indicate significant differences in MAPK activity ($p < 0.05$, $p < 0.01$ and $p < 0.001$, respectively) compared to the 0-min time point

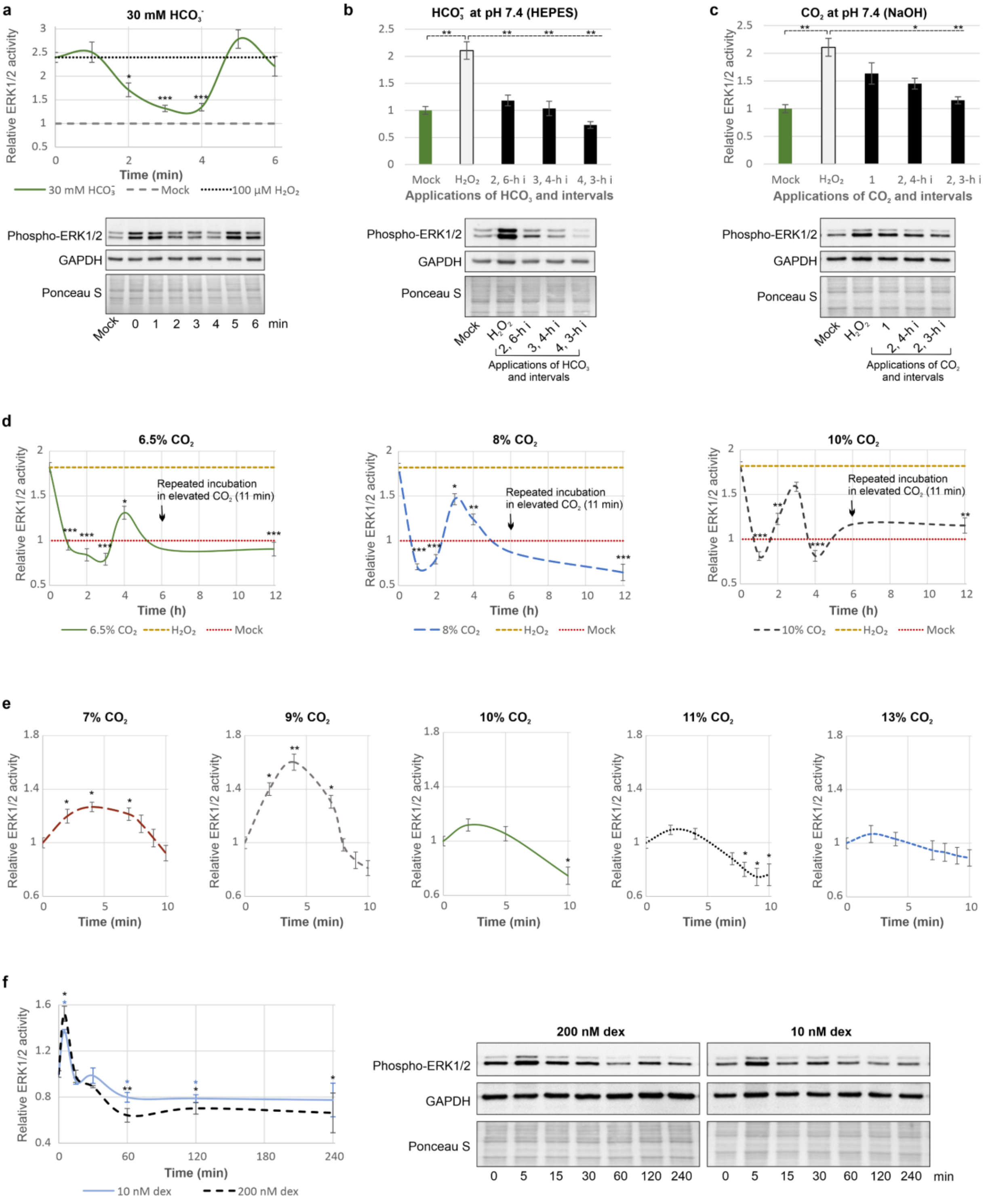

Fig. S2 ERK1/2 activated by 100 µM H_2O_2 are inhibited by both HCO_3^- and CO_2 in ECs. **a** At 30 mM, $NaHCO_3$ transiently inhibited ERK1/2. **b** Repeated treatments, i.e. 2, 3 or 4 incubations as indicated, with 15 mM HCO_3^- buffered with HEPES (pH 7.4) inactivated ERK1/2; i – interval between applications. **c** Neutralised 15 mM CO_2 (cell culture medium containing 14 mM NaOH was bubbled with carbon dioxide until the pH reached 7.4) inactivated ERK1/2.

This effect was enhanced by additional CO_2 applications. **d** Inhibition of ERK1/2 was triggered by 11-min incubations with CO_2 at the indicated concentrations. **e** Time course of H_2O_2-induced ERK1/2 activity in response to CO_2 at the indicated levels. **f** Time course of ERK1/2 activity in response to 10 and 200 nM dex in ECs. In **a–f**, ERK1/2 activity was assessed by immunoblotting with an anti-phospho-ERK1/2 antibody. Protein loading was visualised by Ponceau S staining and immunoblotting with an anti-GAPDH antibody

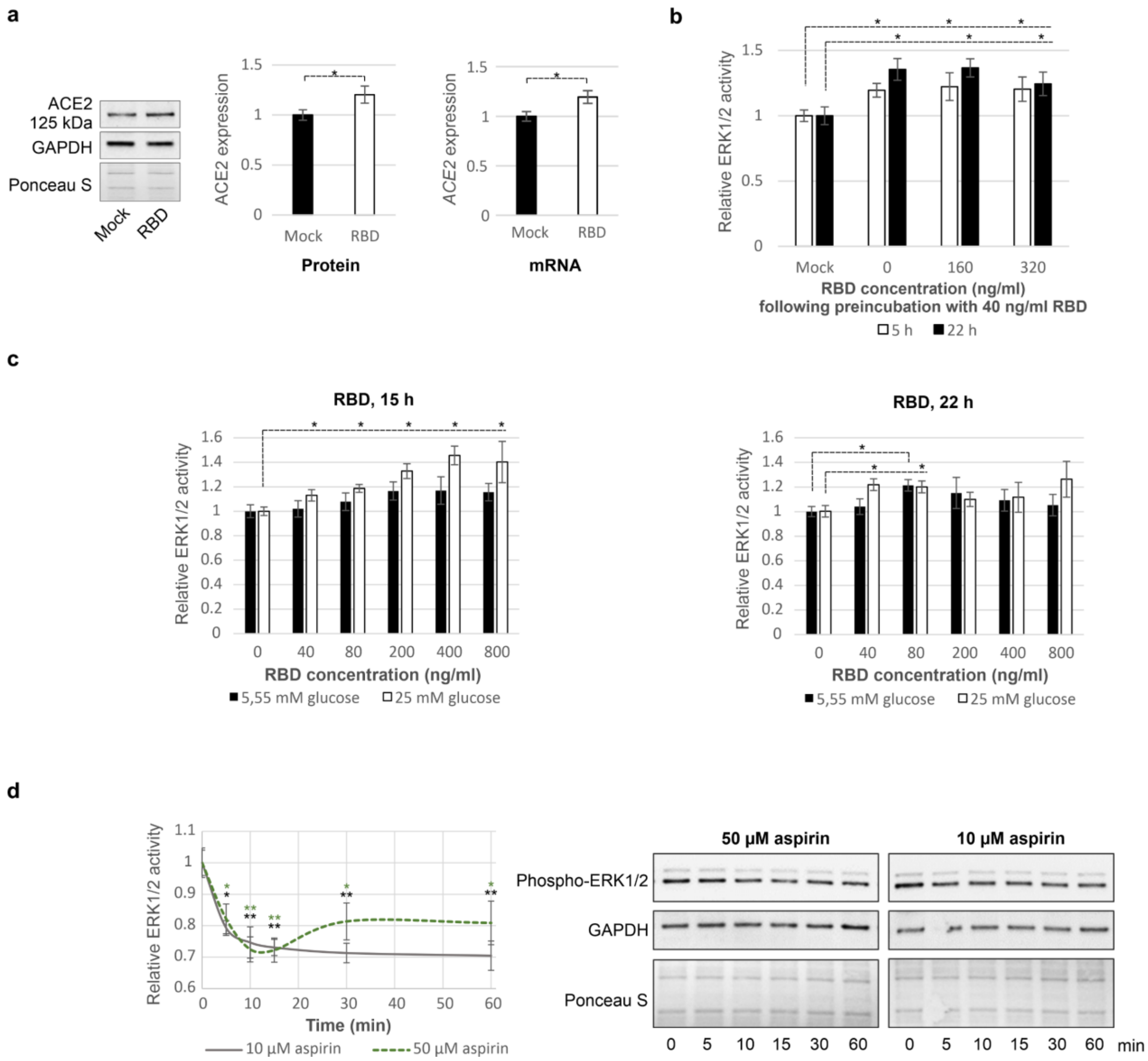

Fig. S3 Experiments supporting RBD-dependent activation of ERK1/2 in ECs.

a Expression of ACE2 in ECs was induced in response to treatment with 40 ng/ml RBD for 24 h. Immunoblotting was performed with anti-ACE2 and anti-GAPDH antibodies. *ACE2* mRNA levels were determined by RT–qPCR with *GAPDH* and *ACTB* as normalization controls. Data from three independent experiments; three technical replicates per experiment. **b** ERK1/2 activity was increased under conditions designed to mimic an increase or decrease in the amount of SARS-CoV-2 over time, i.e. initial incubation with 40 ng/ml RBD for 15 h followed by a change in the RBD concentration (0, 160, 320 ng/ml) and further incubation for 5 or 22 h. Both an increase in concentration and the removal of RBD maintained ERK1/2 activation, unlike the absence of RBD. **c** Effect of glucose concentration on ERK1/2 activation by RBD at the indicated concentrations. **d** Time course of ERK1/2 inactivation by 10 and 50 μM aspirin. In **b–d** ERK1/2 activity was determined by immunoblotting with an anti-phospho-ERK1/2

antibody, and protein loading was assessed by Ponceau S staining and immunoblotting with anti-GAPDH and anti-ERK1/2 antibodies

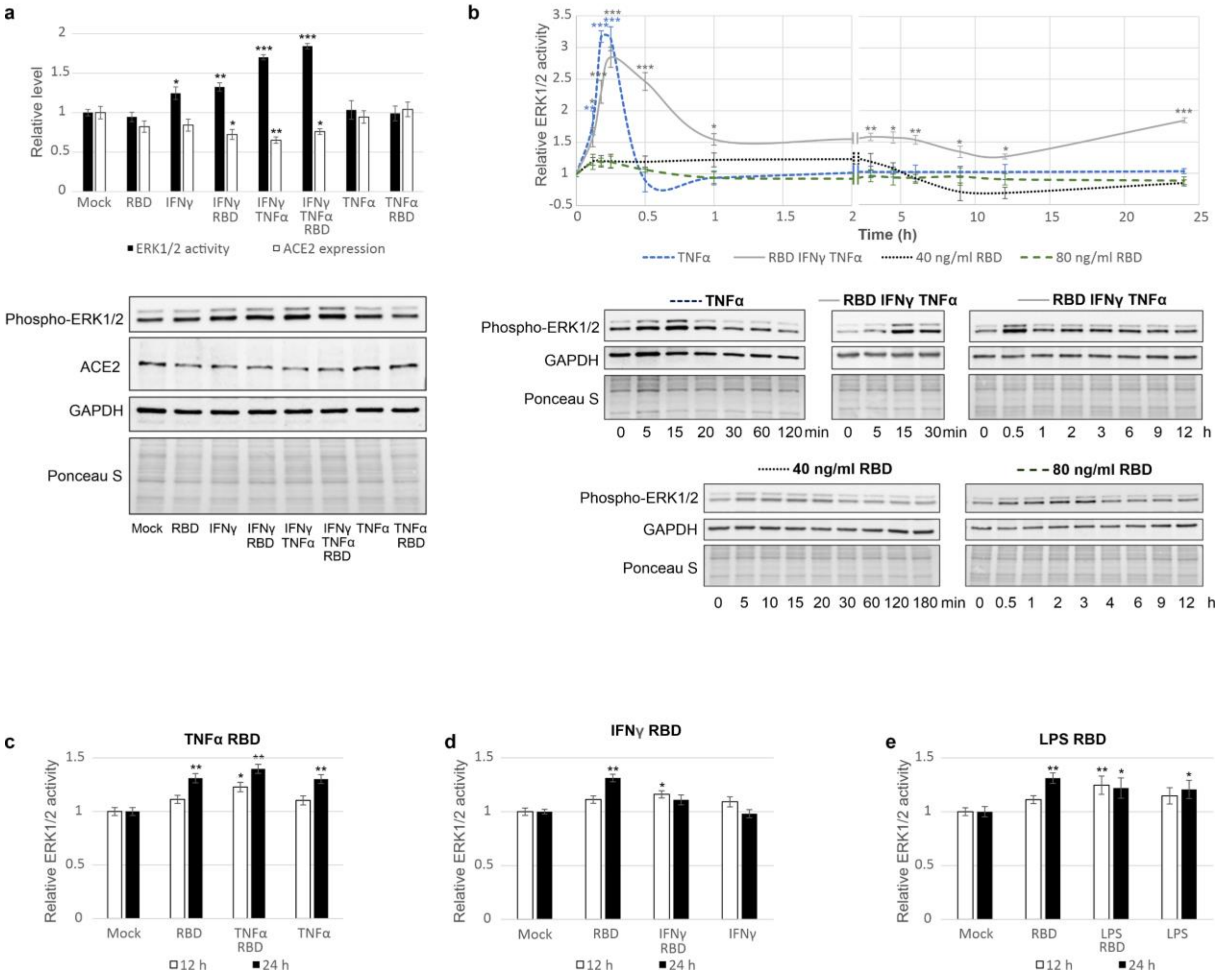

Fig. S4 Effects of proinflammatory cytokines on RBD-induced ERK1/2 activation. **a** RBD and proinflammatory cytokines regulate ERK1/2 activity and ACE2 expression in BEAS-2B cells. Cells were treated with the indicated proteins for 24 h. **b** Time course of ERK1/2 activity in response to RBD, TNFα or the combination of RBD, TNFα and IFNγ in BEAS-2B cells. **c–e** Effect of TNFα (**c**), IFNγ (**d**) and LPS (**e**) combined with 40 ng/ml RBD on ERK1/2 in ECs. In **a–e**, ERK1/2 activity was measured by immunoblotting with an anti-phospho-ERK1/2 antibody. Protein loading was visualised by Ponceau S and immunoblotting with an anti-GAPDH antibody. ACE2 expression in **a** was measured with an anti-ACE2 antibody
